# Mental health outcomes of a pilot 2-arm randomized controlled trial of a HIV-prevention program for South African adolescent girls and young women and their female caregivers

**DOI:** 10.1186/s12889-021-12010-1

**Published:** 2021-11-30

**Authors:** Geri Donenberg, Katherine G. Merrill, Millicent Atujuna, Erin Emerson, Bethany Bray, Linda Gail Bekker

**Affiliations:** 1 Center for Dissemination and Implementation Science, Department of Medicine, University of Illinois at Chicago, 818 S. Wolcott, Chicago, IL 60612 USA; 2 Desmond Tutu HIV Center, Cape Town, South Africa

**Keywords:** Mental health, HIV prevention, South Africa, Adolescent girls and young women, Mother-daughter intervention

## Abstract

**Background:**

South African adolescent girls and young women (AGYW) report significant mental distress and sexual and reproductive health concerns. Mental health problems and trauma symptoms are consistently associated with sexual and reproductive health behavior. Despite their intersection, few interventions address them simultaneously or engage female caregivers (FC) as collaborators. This study presents findings from a pilot test of an empirically supported culturally adapted family-based HIV-prevention program, **I**nformed **M**otivated **A**ware and **R**esponsible **A**dolescents and Adults- South Africa (IMARA-SA), on AGYW anxiety, depression, and trauma.

**Methods:**

Sixty 15–19-year-old AGYW (mean age = 17.1 years) and their FC from outside Cape Town were randomized to IMARA-SA or a health promotion control program. AGYW reported their anxiety using the GAD-7, depression using the PHQ-9, and trauma using the PC-PTSD-5 at baseline and follow-up (6–10 months post). Both interventions were delivered by Xhosa-speaking Black South African women in groups over 2 days for approximately 10 h. We examined intervention effects using zero-inflated negative binomial regression for anxiety, multinomial logistic regression for depression, and logistic regression for trauma.

**Results:**

At baseline, groups did not differ in demographic characteristics but AGYW randomized to IMARA-SA had higher depression scores than controls (*p* = 0.04) and a greater proportion screened positive for PTSD (*p* = .07). Controlling for baseline mental health scores, AGYW who received IMARA-SA compared to controls had significantly fewer anxiety symptoms at follow-up (adjusted incidence rate ratio for count model = 0.54, 95% CI = 0.29–0.99, *p* = 0.05), were less likely to report at least one depressive symptom relative to no symptoms (relative risk ratio = 0.22, 95% CI = 0.05, 0.95, *p* = 0.04), and were less likely to report symptoms of PTSD relative to no symptoms, but this difference was not statistically significant.

**Conclusions:**

Mental health is implicated in risky sexual behavior, and reducing emotional distress can mitigate exposure to poor sexual and reproductive health outcomes. This pilot study yielded promising findings for the mental health impact of IMARA-SA, justifying replication in a larger randomized trial.

**Trial registration:**

ClinicalTrials.gov Number NCT04758390, accepted 17/02/2021.

## Background

Serious emotional distress is a public health crisis for young people worldwide. Estimates indicate that 15% of youth globally experience mental health disorders [1], with depression and anxiety as leading concerns among adolescents [2]. In South Africa, the prevalence of adolescent mental health disorders such as mood, anxiety, and post-traumatic stress ranges from 15 to 41% [3], with upwards of 39% in the Western Cape [4]. Still, stark gender differences exist; adolescent girls and young women (AGYW) are disproportionately affected compared to males [5]. Despite these alarming trends, mental health distress in young South Africans is often underdiagnosed and undertreated [6], amplifying the urgent need for effective and scalable interventions.

Like mental health, AGYW experience more negative sexual and reproductive health (SRH) outcomes than males. South African AGYW are three times more likely to acquire HIV [7] and have higher rates of sexually transmitted infections (STI) [8] despite advances in prevention technologies (e.g., pre-exposure prophylaxis) and decreased incidence in other populations. Several factors are implicated in AGYW’s poor SRH outcomes, including limited access, availability, and uptake of relevant services (e.g., pregnancy prevention, comprehensive sex education) [9]. Innovative approaches are therefore essential to broaden dissemination efforts and reach AGYW in greatest need.

Mental health and SRH intersect in important ways, with well-documented links between emotional distress and poor SRH [10]. Research implicates anxiety, depression, and trauma as drivers of STI/HIV acquisition in South African adolescents [11]. Untreated mental health disorders and lack of available SRH services has led to significant morbidity and mortality, more unwanted pregnancies, and increased STI/HIV among AGYW [12]. There is widespread agreement that interventions that address the intersection of SRH and mental health have the potential to achieve better AGYW health outcomes [10].

The need for co-occurring mental health and SRH services in South Africa far outweighs access and availability [13]. Few evidence-based mental health programs incorporate SRH, and few SRH interventions screen for or integrate mental health [10, 14]. Additional challenges include the scarcity of professionally trained mental health service providers,especially for young people [15], and the absence of adolescent-friendly SRH clinics [16]. Finally, several implementation characteristics serve as barriers to uptake and sustainability of evidence-based programs in low- and middle-income countries, including an absence of health care provider training, mental health stigma, and limited financial resources.

In South Africa, most SRH and mental health programs focus on the individual AGYW. This approach misses an opportunity to leverage meaningful support systems that might strengthen AGYW mental health resilience and positive SRH decision-making. One relatively untapped natural resource is the female caregiver, defined broadly as an important figure in the care of AGYW’s growth and development. Engaging female caregivers in AGYW mental health and SRH is culturally congruent with South African norms. Female caregivers, and aunts specifically, bear most of the responsibility for educating young women about their sexual and reproductive health among Black South Africans. Female caregivers may be able to encourage and strengthen AGYW healthy sexual-decision making, improve uptake of effective HIV/STI prevention technologies (e.g., condom use, pre-exposure prophylaxis (PrEP)), and support learning and application of effective emotion regulation strategies to build resilience.

Evidence from the United States and Africa supports a positive role for caregivers in AGYW mental health and SRH. Studies indicate that family acceptance, parental warmth and monitoring, and strong parental attachment are related to fewer adolescent mental health problems, especially for girls [17]. For South African adolescents, fewer mental health problems have been associated with better parent-adolescent communication [18]. Likewise, female caregivers impact AGYW SRH, whereby less sexual risk taking among AGYW is associated with strong parent-adolescent relationships [19], parental monitoring [20], and mother-daughter communication that is open, receptive, and comfortable [21, 22].

Evidence suggests that family-based interventions within the South African context have shown positive effects on the mental health of adolescents living with and affected by HIV [23–25], including female caregivers in adolescent HIV/STI prevention is associated with lower STI incidence and mental health distress in Black AGYW in the United States. Informed Motivated Aware and Responsible Adults and Adolescents (IMARA) is an evidence-based mother-daughter HIV-prevention intervention developed and evaluated with 14–18-year-old U.S. Black AGYW and their female caregivers. IMARA addresses SRH and teaches emotion regulation strategies to manage difficult emotions and reduce distress. IMARA was associated with a 43% reduction in AGYW STI incidence at 12-month follow-up compared to a time-matched health promotion program [26] and improved mental health symptoms at 6- and 12-months among girls with high mental health distress at baseline [27]. These findings are promising but may not generalize to other cultural contexts or populations.

From April through October 2019, IMARA was subjected to a rigorous adaptation process involving key stakeholders, an advisory board, AGYW, and caregivers and tailored for South African AGYW and their female caregivers. The revised curriculum (i.e., IMARA-SA) was subsequently tested in a small 2-arm pilot study to evaluate implementation outcomes (feasibility, acceptability) and preliminary effectiveness on sexual risk taking and prevention uptake (e.g., PrEP). In this paper, we report on the pilot study effects of IMARA-SA on AGYW mental health distress, namely anxiety, depression, and trauma, secondary outcomes of the intervention. The study’s primary outcomes (sexual health) are presented elsewhere (Merrill K, Atujuna M, Emerson E, Blachman-Demner D, Bray BC, Bekker L-G, Donenberg G: IMARA SA: piloting a family-based HIV/STI prevention intervention for south African adolescent girls, submitted). We hypothesized that compared to AGYW who received a health promotion control program, AGYW who received IMARA-SA would report less anxiety, depression, and trauma at follow-up.

## Methods

### Participants

The pilot study was implemented during phase one of a two-phased award. AGYW were eligible if they were: a) female; b) Black or mixed race; c) 15–19 years old; d) residing in Klipfontein/Mitchells Plain or neighboring areas; and e) an English and/or Xhosa speaker. Caregivers were eligible if they were: a) female; b) identified by the AGYW as a female caregiver; c) 24 years or older; d) living with or in daily contact with the AGYW; and e) an English and/or Xhosa speaker. Caregivers ranged in age from 24 to 60 years (mean = 36.6 years, SD = 9.4), and most were biological mothers (42%), aunts (27%), or sisters (18%) (Table 1).

**Table 1:** Baseline characteristics of intervention and control groups (*n* = 60 adolescent girls and young women)

	Total	Intervention	Control	***p*** value*
Full sample	60	30	30	N/A
**Demographics**				
Age	17.13 (1.53)	17.23 (1.48)	17.03 (1.61)	0.62
Highest education achieved				
Primary school	22 (36.7%)	11 (36.7%)	11 (36.7%)	0.11
Secondary school	34 (56.7%)	19 (63.3%)	15 (50.0%)	
Higher education	4 (6.7%)	0 (0.0%)	4 (13.3%)	
Supported self financially in past year	10 (16.7%)	3 (10.0%)	7 (23.3%)	0.30^
**Participation**				
Female caregiver participating				
Biological mother	25 (41.7%)	13 (43.3%)	12 (40.0%)	0.69
Aunt	16 (26.7%)	6 (20.0%)	10 (33.3%)	
Sister	11 (18.3%)	7 (23.3%)	4 (13.3%)	
Cousin	5 (8.3%)	3 (10.0%)	2 (6.7%)	
Grandmother	1 (1.7%)	0 (0.0%)	1 (3.3%)	
Other	2 (3.3%)	1 (3.3%)	1 (3.3%)	
**Mental health measures**				
Anxiety				
No symptoms (scores of 0)	9 (15.0%)	5 (16.7%)	4 (13.3%)	0.56
Symptoms of anxiety (scores of 1–9)	36 (60.0%)	16 (53.3%)	20 (66.7%)	
Positive screen for GAD (scores of 10–21)	15 (25.0%)	9 (30.0%)	6 (20.0%)	
Anxiety score (range: 0–21)	6.41 (5.44)	7.47 (5.81)	5.36 (4.92)	0.14
Depression				
No symptoms (scores of 0)	4 (6.7%)	1 (3.3%)	3 (10.0%)	0.18
Symptoms of depression (scores of 1–9)	38 (63.3%)	17 (56.7%)	21 (70.0%)	
Positive screen for depression (scores of 10–27)	18 (30.0%)	12 (40.0%)	6 (20.0%)	
Depression score (range: 0–27)	7.85 (6.01)	9.4 (6.33)	6.3 (5.32)	0.04
PTSD				
No symptoms (scores of 0)	32 (53.3%)	15 (50.0%)	17 (56.7%)	0.07
Symptoms of PTSD (scores of 1–2)	11 (18.3%)	3 (10.0%)	8 (26.7%)	
Positive screen for PTSD (scores of 3–5)	17 (28.3%)	12 (40.0%)	5 (16.7%)	

*Abbreviations*: *GAD* generalized anxiety disorder, *PTSD* post-traumatic stress disorder

Figures are n (%) or mean (SD)

*Chi-square test for binary/categorical variables and t-test for continuous variables

^Fisher’s exact test

### Procedures

AGYW and their caregivers were recruited from October 2019 to January 2020, through street outreach, neighborhood canvassing, word-of-mouth, flyers, and after school programs and clinics at the Desmond Tutu Health Foundation (DTHF). Interested families contacted research staff who explained the project in detail, including information about the trial design (e.g., random assignment) and confidentiality of STI results. Research staff scheduled a baseline assessment that included surveys and clinical data collection (e.g., STI and HIV testing) at DTHF. AGYW and their female caregivers separately reviewed the consent and assent documents with research staff. All measures and materials were translated from English into Xhosa, and participants selected their preferred language for the consent process and surveys.

AGYW and caregivers independently completed a 2-h tablet-based assessment and were offered HIV testing and counseling, STI testing, and PrEP where appropriate. AGYW-caregiver dyads were randomized to conditions (*n* = 30 IMARA-SA; *n* = 30 control) following the baseline survey, and participated in an introduction to IMARA-SA or the control program. Randomization occurred by having AGYW select their program from a paper bag without replacement to ensure equal numbers in each arm. After the introduction, participants completed the clinical data collection. Both participants in the dyad received R130 for their time and transport.

**Fig. 1:**
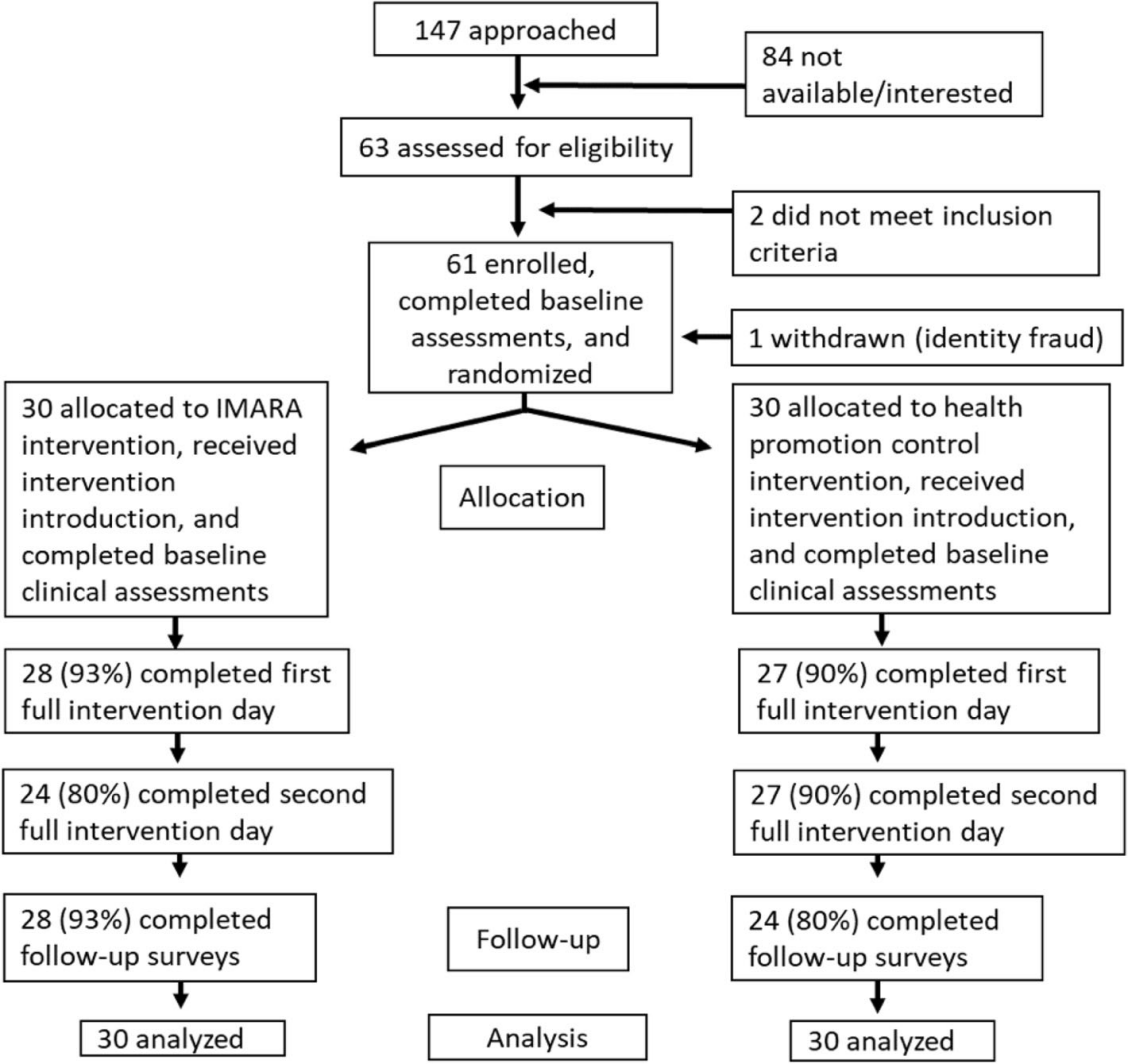
Consort diagram for the participation of adolescent girls and young women in the pilot study

Dyads returned on one or two separate occasions to complete the intervention, for a total of about 10 h of content. Snacks and a meal were provided on intervention days. IMARA-SA and the control program were delivered in groups of two to nine dyads. At the end of each intervention day, AGYW and caregivers separately completed program evaluations, and group leaders and observers rated their adherence to the curriculum. Dyads returned for a follow-up survey between 6- and 10-months post-baseline. The timeframe for follow-up surveys was impacted by the COVID pandemic. Participant retention was strong despite the onset of COVID mid-way through the study; 85% of dyads attended the full intervention and 87% completed the follow-up survey (see Fig. 1 Consort Diagram). The study was approved by the US (2018–0709) and South African (077/2019) ethics committees, and all methods including human participants/data were carried out in accordance with relevant guidelines and regulations.

### Intervention and control program descriptions

#### Overview

**Fig. 2:**
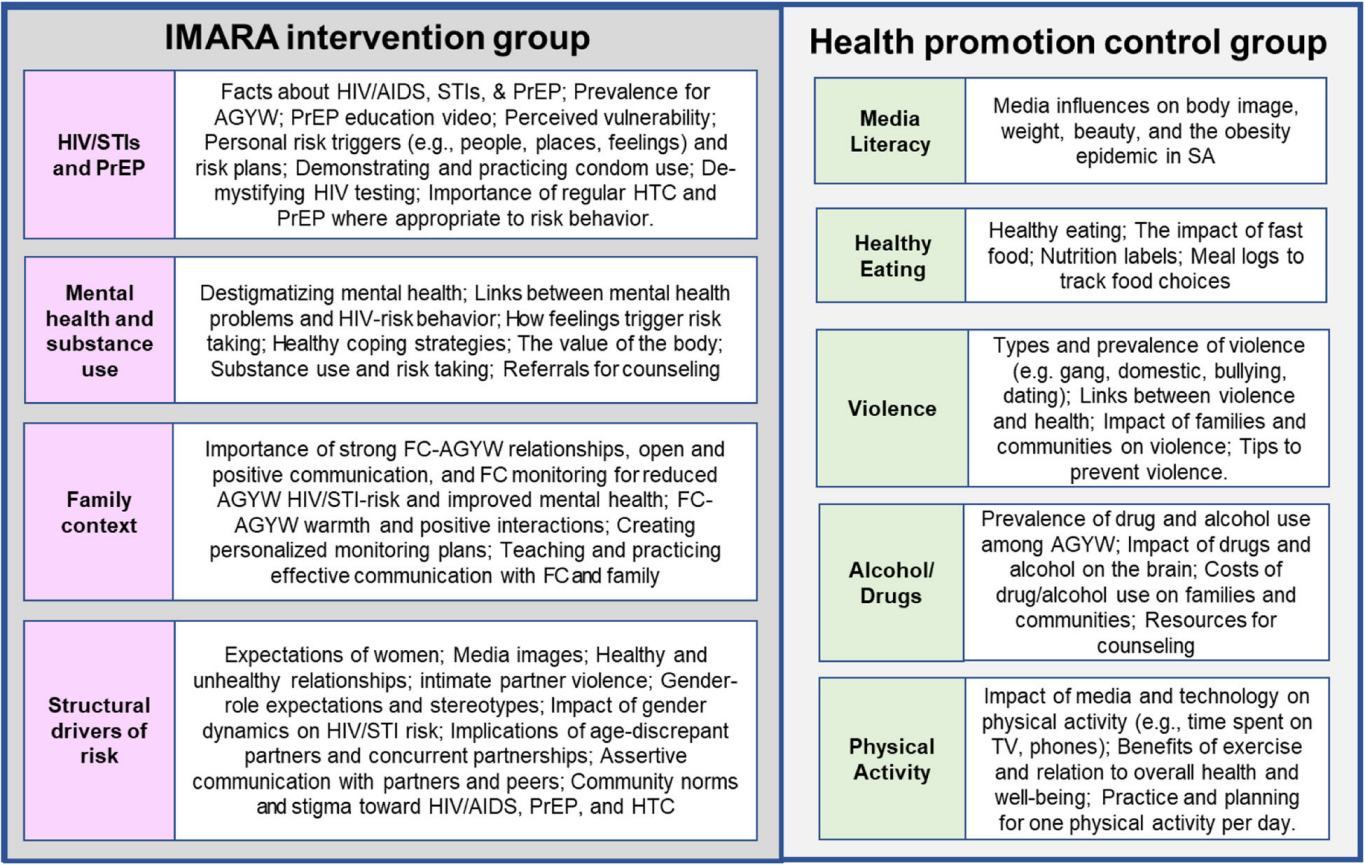
Curriculum content for IMARA and health promotion conditions

IMARA-SA and the health promotion control program were matched for contact time and format. In both interventions, some activities were delivered separately to AGYW and caregivers covering parallel content, and some activities involved AGYW and caregivers together in a single group. Participants in the IMARA-SA and health promotion program did not overlap. For each program, two facilitators led the caregiver group and two led the AGYW group. All four facilitators led the joint activities. Figure 2 lists the activities in each intervention condition.

#### IMARA-SA

The original IMARA program was derived from three evidence-based interventions, and included significant input from stakeholders, community members, and pilot testing [26]. IMARA-SA’s curriculum adopted most of the original content after careful cultural tailoring. IMARA-SA is designed to strengthen AGYW-caregiver relationships and communication about STI/HIV prevention and safer sexual behavior, increase self-efficacy to use condoms, improve caregiver monitoring of AGYW activities, promote pride in South African female culture, and encourage gender empowerment. Caregivers and AGYW learn and practice skills through role-plays and games, discuss healthy and unhealthy relationships, and consider social media’s impact on South African AGYW’s self-image. Caregivers develop plans to monitor AGYW activities, and AGYW identify triggers (e.g., people, places, moods) of risk behavior and create personalized plans to manage them. Interactive and experiential activities (role plays, games) are employed to strengthen caregivers’ credibility as a resource for STI/HIV prevention. Dyads discuss challenging topics to improve conflict negotiation and assertive communication and receive feedback from group members. The curriculum emphasizes the impact of mental distress on SRH and teaches strategies to manage emotions. For example, AGYW and their caregivers are taught to recognize when their emotions are in the “hot zone” because this is when unsafe decisions are more likely to occur. Strategies to “cool off” are discussed and practiced.

#### Control program

The control program emphasizes healthy eating and nutrition, physical activity and exercise, informed consumer behavior, beauty standards for African women and girls, personal and societal facts and costs of drug/alcohol use, and violence prevention. The curriculum is based on a health promotion program used in prior research (Sales J, Renfron T: FUEL: a health promotion program, unpublished), but adapted to ensure topics addressed the most important general health concerns for South African AGYW and their caregivers. Like IMARA-SA, the health promotion curriculum employs an interactive approach, using games, videos, factual presentations by facilitators, and hands-on activities. Dyads practice relaxation and develop physical activity pyramids. Caregivers and AGYW do not engage in role-plays or joint communication exercises, and they do not receive condom skills training.

#### Facilitator training

Facilitators for both programs were Black South African women with and without previous experience leading groups. They did not overlap arms and were trained separately over a month until competency was reached (approximately 30 h total). Training was led by the U.S.-based IMARA team and Principal Investigator and the South African site Co-Investigator using a detailed manual. Training emphasized the importance of manualized interventions, adhering to intervention content, and techniques to encourage participation. Facilitators practiced each activity, conducting “mock run-throughs,” and received feedback until deemed competent by the South African site’s Co-Investigator. Competency was determined by accurate/adherent, clear, and comfortable delivery of the intervention to the trainers and trainees who posed as young women and mothers. Training reviewed the facts of STI/HIV transmission and prevention, AGYW psychosexual development, group dynamics, and behavior management.

#### IMARA-SA Fidelity

Observers rated facilitators’ adherence to each activity at the end of each workshop day. Ratings were reviewed by the South African project coordinator following the intervention sessions and discussed during supervision along with any concerns and need for additional training. Treatment fidelity based on observer reports was 94.8%.

### Outcome measures

#### Anxiety

AGYW completed the 7-item Generalized Anxiety Disorder-7 (GAD-7) [28] to indicate the frequency of anxiety symptoms in the past 2 weeks on a scale from 0 = not at all to 3 = nearly every day (score range = 0–21). Scores of 10 or greater are considered clinically significant and represent a diagnosis of GAD. The GAD-7 has strong construct validity, internal consistency, and test-retest reliability [28], including in African settings [29]. The measure demonstrated good internal consistency among AGYW in this study (α = 0.85) [30]. We examined anxiety as both a continuous and categorical measure. The categorical variables assessed the numbers and proportions of participants with no symptoms (scores of 0), symptoms of anxiety (scores of 1–9), and a positive screen for GAD (scores of 10 or greater).

#### Depression

AGYW completed the 9-item Patient Health Questionnaire (PHQ-9) [31] to indicate the frequency of depression symptoms in the past 2 weeks on a scale from 0 = not at all to 3 = nearly every day (range = 0–27). The PHQ-9 has demonstrated reliability for screening depression in adults in South Africa [32] and other African settings [33, 34] and is recommended for adolescents [35]. The measure showed acceptable internal consistency among AGYW in this study (α = 0.78) [30]. A score of 10 or more indicates clinical levels of depression in validation studies [32, 33], with good test**–**retest reliability in Ethiopia [33] and Kenya [34]. We evaluated depression as both a continuous and categorical measure. The categorical variables indicated the numbers and proportions of participants with no symptoms (scores of 0), symptoms of depression (scores of 1 to 9), and a positive screen for depression (scores of 10 or higher). The suicidal ideation question was omitted at follow-up due to lack of staffing to support suicidal youth during COVID. Hence, the score range at follow-up was 0–24.

#### Trauma

AGYW completed the Primary Care PTSD Screen for *DSM-5* (PC-PTSD-5) [36]. This measure has been widely used in low resource countries including Zambia with good reliability (Cronbach**’**s alpha **≥**0.90) [37] and Kenya [38]. The first item asks about any lifetime exposure to a traumatic event. If the respondent reports none, she was assigned a score of 0 and receives no further questions. If the AGYW indicated an exposure, she completed five additional yes/no questions about the influence of the trauma in the past month. Preliminary validation studies indicate a positive screen for PTSD with a score of three or more. We created a categorical measure for the analysis as follows: no symptoms (scores of 0), symptoms of PTSD (scores of 1 to 2), and a positive screen for PTSD (scores of 3–5).

### Data analyses

We used an intent-to-treat analysis to examine intervention effects on mental health outcomes. We compared demographic and outcome variables between groups at baseline using chi-square tests for binary/categorical variables and t-tests for continuous variables. We used chi-square tests and t-tests to examine differences between AGYW lost-to-follow-up and those who completed follow-up.

Group differences in anxiety at follow-up were evaluated using zero-inflated negative binomial regression due to the positive skew in the distribution and the presence of excessive zeros (i.e., no reports of symptoms) at follow-up. This regression approach has two components: 1) a logistic regression model to predict the odds of excess zeros, and 2) a negative binomial count model to predict the rate of anxiety symptoms. We obtained crude and adjusted odds ratios (ORs) for Component 1 and incidence rate ratios (IRRs) for Component 2. Adjusted models controlled for baseline anxiety scores. We confirmed our model selection using goodness of fit criteria (e.g., Akaike information criteria). To evaluate group differences in depression at follow-up, we used multinomial logistic regression, treating depression as a categorical variable, given the positive skew and relatively flat shape of the distribution at follow-up. We obtained a crude relative risk ratio (RRR) and an adjusted RRR which controlled for baseline depression score. Given the small number of AGYW who reported PTSD symptoms at follow-up, we collapsed the categorical variable into a binary variable to assess no symptoms of PTSD (scores of 0) versus any symptoms of PTSD (scores of 1–5) at follow-up. We compared group differences by generating crude and adjusted ORs using logistic regression. The adjusted model controlled for the presence or absence of PTSD symptoms at baseline. Although the interventions were implemented in groups, AGYW did not remain with the same participants for all sessions. Thus, analyses did not need to account for possible clustering of participants. Analyses were conducted using STATA 15.

## Results

Of 60 AGYW who completed the baseline assessment, 52 (87%) returned for follow-up. AGYW were balanced across conditions on baseline demographic characteristics, and AGYW lost to follow-up did not differ from those retained on baseline demographic or mental health characteristics. At baseline, AGYW were on average 17.1 years old (SD = 1.53) (Table 1), about one third had completed primary school (37%), and over half had completed secondary school (57%). About one-sixth (17%) had supported themselves financially in the past year. Almost all AGYW reported living in homes with a cellphone (98%) and electricity (92%); 63% were in homes with a refrigerator and 20% with a personal computer. Over half (57%) of AGYW reported living with the female caregiver who participated in the study. Regarding mental health, 93% of AGYW reported at least one symptom of depression, 85% reported at least one symptom of anxiety, and 47% reported at least one symptom of PTSD (Table 1). Groups were generally balanced on baseline mental health variables with two exceptions. IMARA-SA AGYW had higher scores for depression (9.4 vs. 6.3 in the control group, *p* = 0.04), and there was weak evidence of a difference in PTSD symptoms at baseline (*p* = 0.07); a greater proportion of IMARA-SA AGYW had a positive screen for PTSD (40%) compared to control group participants (17%).

Across all outcomes, reductions in symptoms were observed in both the IMARA-SA and control groups between baseline and follow-up. IMARA-SA AGYW had significantly fewer anxiety symptoms at follow-up compared to control AGYW, controlling for baseline anxiety score (adjusted IRR for count model: 0.54, 95% CI = 0.29, 0.99, *p* = 0.05) (Table 2). IMARA-SA AGYW were also less likely than the control group to have depressive symptoms (i.e., scores of 1–9) relative to no symptoms (i.e., scores of 0), controlling for baseline depression score (RRR: 0.22, 95% CI: 0.05, 0.95, *p* = 0.04). The effect for a positive screen for depression (i.e., scores of 10 or more) favored the IMARA-SA group but was non-significant (Table 3). Compared to the control group, IMARA-SA AGYW were less likely to have symptoms of PTSD relative to no symptoms at follow-up, but this difference did not reach statistical significance (Table 3).

**Table 2:** Results from zero-inflated negative binomial regression for differences in anxiety symptoms between treatment groups at follow-up

	Probability of having zero symptoms: Logistic model						Probability of anxiety: Count model					
	Crude Odds Ratio	95% CI	***p*** value	Adjusted Odds Ratio	95% CI	***p*** value	Crude Incidence Rate Ratio	95% CI	***p*** value	Adjusted Incidence Rate Ratio	95% CI	***p*** value
Treatment group (intervention vs. control)	1.04	(0.97, 1.11)	0.29	1.04	(0.98, 1.11)	0.25	0.76	(0.39, 1.46)	0.41	0.54	(0.29, 0.99)	0.05

*Adjusted estimates control for anxiety score at baseline

**Table 3:** Differences in depression and post-traumatic stress disorder (PTSD) symptoms between treatment groups at follow-up

	Intervention group		Control group		Crude estimate	95% CI	***p*** value	Adjusted estimate^**a**^	95% CI	***p*** value
	Baseline (***n*** = 30)	Follow-up (***n*** = 28)	Baseline (***n*** = 30)	Follow-up (***n*** = 24)						
Depression										
No symptoms (scores of 0)	1 (3.3%)	11 (39.3%)	3 (10.0%)	4 (16.7%)	1			1		
Symptoms of depression (scores of 1–9)	17 (56.7%)	13 (46.4%)	21 (70.0%)	16 (66.7%)	0.30	(0.08, 1.14)	0.08	0.22	(0.05, 0.95)	0.04
Positive screen for depression (scores of 10+)^b^	12 (40.0%)	4 (14.3%)	6 (20.0%)	4 (16.7%)	0.36	(0.06, 2.19)	0.27	0.17	(0.02, 1.52)	0.12
PTSD										
No symptoms (scores of 0)	15 (50.0%)	22 (78.6%)	17 (56.7%)	18 (75.0%)	1			1		
Symptoms of PTSD (scores of 1–5)	15 (50.0%)	6 (21.4%)	13 (43.3%)	6 (25.0%)	0.82	(0.22, 2.98)	0.76	0.85	(0.21, 3.27)	0.81

Estimates are relative risk ratios (RRRs) for depression and odds ratios (ORs) for PTSD

^a^Adjusted estimates control for the outcome at baseline

^b^Score range is 0–27 at baseline and 0–24 at follow-up since one item assessing suicidal thoughts was removed from the follow-up survey

## Discussion

Untreated emotional distress has profound and negative effects on individual well-being, community health, and national productivity, particularly in the context of poverty and other vulnerabilities. South African AGYW are at increased risk for significant mental health problems, but few evidence-based interventions leverage family support and simultaneously address SRH in the South African context. This study is among the few to demonstrate the preliminary effects of an evidence-based culturally tailored SRH and mental health program that includes female caregivers on AGYW anxiety, depression, and trauma. Even with a small sample size, AGYW who received IMARA-SA reported significant reductions in two of the three types of mental health problems at follow-up, and the third trended in the desired direction.

Findings are particularly salient in the context of the COVID pandemic. COVID-19 has led to increased social isolation, loneliness, and lack of connection, especially among adolescents who have been separated from peers, unable to attend school, and quarantined in their homes [39, 40]. These conditions have amplified pre-existing mental health distress in young people, and exacerbated feelings of despair and hopelessness. This study in which follow-up data collection occurred after the onset of COVID-19 revealed reductions in all three mental health problems across groups and significantly greater reductions in two of the three types among AGYW who received IMARA-SA. It is possible that IMARA-SA strengthened the AGYW-caregiver bond such that it has protected, at least in part, against the isolation and loneliness bought on by the pandemic. Likewise, with renewed attention to improving adolescent mental health, IMARA-SA may be a viable option as young people look to their families for support in the current climate. Additional research is needed to explore this possibility.

An important feature of IMARA-SA is that it can be delivered by individuals without prior experience in mental health service delivery or educational attainment as long as they are properly trained and supervised. Task-shifting—i.e., employing trained lay health workers to deliver care in non-specialist settings [41]—has been identified as an important approach to address the urgent need for mental health services in low- and middle-income countries [42]. Despite implementation barriers to task shifting, efforts have been effective and cost-effective [41]. By not relying on professionally trained mental health experts to deliver IMARA-SA, it is possible that a larger workforce of individuals could be trained to address the scarcity of mental health resources. Task shifting can also allow care to be delivered in diverse settings outside a traditional clinic, such as schools, work places, and community centers. Such settings might be ideal for IMARA-SA where families congregate, if findings are replicated in a larger randomized trial.

Elucidating the underlying mechanisms by which IMARA-SA impacts AGYW mental health is an important direction for future research. For example, there is evidence that depressive symptoms in AGYW are associated with decreased assertiveness in sexual relationships and an inability to withstand social pressure like peer pressure to engage in risky sexual activity. By strengthening AGYW-FC relationships, IMARA-SA may enhance AGYW’s ability to withstand social pressures and be less vulnerable to intimate partner violence and abuse. Likewise, IMARA-SA may increase feelings of social support, decrease mental health stigma, and help AGYW view female caregivers as credible resources. These hypotheses are important to test in future studies with larger samples.

Study limitations merit careful consideration of the results. The present pilot findings may not generalize beyond South African AGYW. This preliminary test of IMARA-SA engaged a small sample that, although likely representative of the target population, requires replication before definitive conclusions can be drawn. We deliberately enrolled different female caregiver types in the study based on feedback during the adaptation phase. It is possible that allowing AGYW and mothers to select the most culturally appropriate female family member may have impacted the patterns observed. Future research should evaluate potential effects of caregiver type on AGYW outcomes. The onset of COVID occurred after the intervention was complete, but before the follow-up assessments. Hence, findings from the follow-up interviews may reflect unique circumstances related to COVID. Future research should be able to distinguish whether effects were specific to the pandemic.

## Conclusions

This is the first investigation of IMARA-SA, and preliminary findings support its efficacy in reducing AGYW anxiety, depression, and trauma. If findings are replicated in the phase-2 clinical trial and effects emerge for sexual health behavior, the key next steps will be to clarify the contexts in which IMARA-SA can be delivered and whether it should be brought to scale. The application of implementation science and the use of qualitative inquiry with implementers and participants will provide guidance on how to identify the factors that both impede and facilitate uptake, adoption, and sustainment.

## Data Availability

The datasets used and analysed during the current study are available from the corresponding author on reasonable request.

## Abbreviations

AGYW: Adolescent Girls and Young Women
FC: Female Caregivers
SA: South Africa
GAD-7: Generalized Anxiety Disorder – 7
PHQ-9: Patient Health Questionnaire – 9
PC-PTSD-5: Primary Care PTSD Screen for *DSM-5*
PTSD: Post Traumatic Stress Disorder
SRH: Sexual and Reproductive Health
HIV: Human Immunodeficiency Virus
PrEP: Pre-Exposure Prophylaxis
US: United States
RRR: Relative Risk Ratio
IRR: Incident Rate Ratios
DTHF: Desmond Tutu Health Foundation
IMARA: Informed, Motivated, Aware, and Responsible Adolescents and Adults
IMARA-SA: Informed, Motivated, Aware, and Responsible Adolescents and Adults – South Africa
STI: Sexually Transmitted Infection
